# Fast Analysis of Multilayer Micro-Machined Coupler Based on Mode-Matching Method

**DOI:** 10.3390/mi17040412

**Published:** 2026-03-27

**Authors:** Sheng Li, Yun Zhao, Hao Gu, Shisheng Yang, Zhongbo Zhu, Chongdi Duan, Tingting Wang, Shengxiao Jin, Caixia Wang, Wei Shao, Jiangqiao Ding

**Affiliations:** 1 National Key Laboratory of Science and Technology on Space Microwave, China Academy of Space Technology Xi’an Branch, Xi’an 710100, China; yangsc0309@163.com (S.Y.); 504.cast@163.com (Z.Z.); duanchongdi@hotmail.com (C.D.); ttw14121046@163.com (T.W.); shengxiao_jin@163.com (S.J.); wangcx504@163.com (C.W.); shaowei_cast504@126.com (W.S.); 2 School of Electronic and Information Engineering, Nanjing University of Information Science and Technology, Nanjing 210044, China; zhyun_922@163.com (Y.Z.); guhao_2315@163.com (H.G.); dingjiangqiao@126.com (J.D.)

**Keywords:** directional coupler, DRIE, mode-matching method (MMM), terahertz (THz), waveguide

## Abstract

The development of next-generation terahertz (THz) transmitters and receivers based on 3D stacked packaging technology relies heavily on the integration of high-performance waveguide directional couplers. This paper presents an accurate and efficient method based on the mode-matching method (MMM) for the rapid analysis of a branch waveguide coupler fabricated through a silicon-based 3D stacking process. In contrast to the traditional method using the finite-element method (FEM) in HFSS, which is cumbersome and time-consuming, the proposed method offers orders-of-magnitude speed improvement. It is especially well-suited for large-scale uncertainty error analysis and statistical evaluation of THz waveguide couplers and related components. This theoretical MMM is validated through an experiment by characterizing a deep reactive ion etching (DRIE) fabricated and 3D stacked 220 GHz waveguide coupler.

## 1. Introduction

The terahertz (THz) frequency band is generally defined within the range of 0.1–10 THz, spanning short millimeter waves, submillimeter waves, and far-infrared wavelengths. As a critical interdisciplinary frontier, it demonstrates outstanding application potential in fields such as radio astronomy and wireless communication [1,2,3], particularly due to its penetrability, which enables communication through plasma blackouts during spacecraft reentry [4]. The interest in next-generation and multi-functional THz instruments and systems has recently attracted significant attention, leading to a high demand for essential THz devices and components, such as sources [5], amplifiers [6], mixers [7], antennas [8], filters [9] and hybrid couplers. For most THz circuits, the rectangular waveguide, known for its low loss, simple fabrication, and efficient interconnection, is the preferred solution [5,6,7,8,9,10,11]. Next-generation THz waveguide-type systems are expected to integrate modules through three-dimensional (3D) stacked micro-machined packaging technology [7,8,9,10,11]. Waveguide couplers, fundamental building blocks for power combining, dividing, and isolation, have sparked strong global research interest and become essential components in applications such as power-combined multipliers [5,10], sideband separating (2SB) superconductor-insulator-superconductor (SIS) mixers [7], and multi-pixel receivers [11,12].

To date, a variety of THz waveguide coupler designs have been developed to meet diverse performance requirements. These include traditional branch waveguide couplers [13], modified branch couplers with easy fabrication [14], wideband couplers with ultra-low amplitude imbalance [15,16], ultra-wideband couplers employing symmetric waveguide widening to achieve low amplitude imbalance [17], highly balanced interdigital hybrid metallic couplers [18], and integrated couplers with polarized rotation [19]. Although various characteristics of THz couplers have been reported, precise dimensions and tight tolerances are required [15,16,17,18,19], posing challenges for fabrication using standard computer numerical control (CNC) milling techniques. As a result, existing devices are largely limited to operation within the W-band, with the highest frequency currently reaching the WR-4.3 band. A promising approach for the THz band is the adoption of micro-machining technologies, such as deep reactive ion etching (DRIE), to realize coupling structures with the required precision [20]. However, traditional planar-type fabrication suffers from limited practical compact applicability due to the requirement for additional long waveguides for input/output connections, as well as issues such as wafer fragility and difficulties in assembly and usage. Therefore, the development of highly integrated THz waveguide couplers based on 3D stacked micro-machining and packaging is particularly valuable. A major emerging challenge in this context is the rapid analysis and statistical evaluation of errors between different layers. Although refined methods exist for determining initial dimensions [21] and a mode analysis approach to estimate the coupling ratio of a symmetrical four-port branch waveguide coupler [22] have been developed, they remain unsuitable for rapid error analysis, particularly concerning etching depth variations, silicon wafer thickness tolerances, and bonding misalignment errors in multilayer architecture couplers.

In this work, an accurate and efficient analysis approach based on the mode-matching method (MMM) is proposed for evaluating a branch waveguide coupler fabricated through a 3D silicon-based stacking packaging process. Traditional MMM applications mainly focus on analyzing single discontinuities or simple waveguide components, such as irises, filters, and junctions. Although suitable for initial design approximations, traditional methods do not offer the modular architecture required for 3D stacked structures and often prove to be computationally demanding. In contrast, the proposed method models the complete stacked coupler by cascading reusable three-port and two-port generalized scattering matrix blocks. This makes the approach especially suitable for multilayer architectures, where layer-dependent fabrication and assembly errors can be incorporated directly and evaluated efficiently. Consequently, the method offers a distinct advantage for rapid uncertainty and statistical analysis of stacked waveguide structures. The waveguide branch coupler is treated as a cascade of three-port waveguide discontinuities. This MMM is implemented by cascading the generalized S-parameters of the discontinuous waveguide structures, resulting in extremely short computing time. While simplified techniques like equivalent circuit approaches provide fast preliminary estimations, they struggle to accurately model the complex multi-mode interactions and thick-iris parasitic effects in densely stacked sub-THz structures. The proposed MMM framework overcomes this by retaining rigorous full-wave accuracy. Unlike the Finite Element Method (FEM), MMM mathematically bypasses computationally expensive 3D volumetric meshing. This speed advantage over FEM makes large-scale Monte Carlo statistical evaluations of fabrication tolerances feasible, a task that remains computationally prohibitive for traditional FEM solvers. To validate this theoretical work, a 220 GHz five-branch waveguide coupler fabricated using DRIE process and packaging with 3D stacking technology is calculated, measured, and analyzed for fabrication errors.

## 2. MMM for Coupler

### 2.1. Discontinuous Structures of Couplers

**Figure 1:**
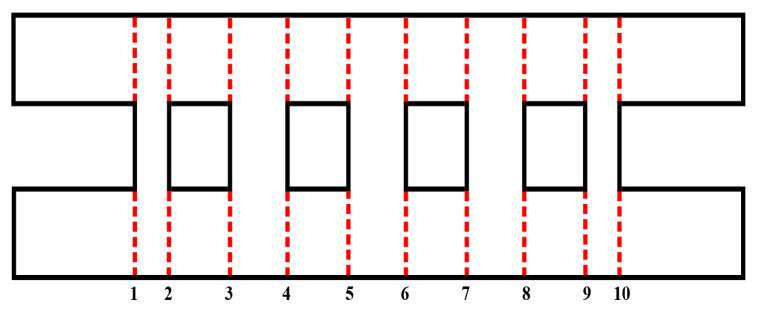
Plane view and discontinuous structures division of a traditional five-branch coupler.

As is well known, the MMM is particularly suited for analyzing abrupt discontinuities in waveguides. It employs analytical methods to rapidly and accurately compute the scattering parameters of discontinuous structures, utilizing the generalized scattering matrix (GSM) approach to derive the overall response. Taking a five-branch waveguide directional coupler structure as an example, the first step is to partition the structure into discontinuous sections, as shown in Figure 1. The discontinuities (1, 2, 3…) in Figure 1 can be regarded as three-port waveguide discontinuities with two input ports and one output port. Other structures, like the waveguide segment between surfaces 1 and 2, can be regarded as the cascade of a two-port network. Therefore, the overall coupler structure can be simplified as multiple cascades of three-port discontinuous structure matrices. The analysis response of the entire coupler can ultimately be achieved with the help of the GSM method.

### 2.2. MMM Analysis for Multiport Structure

**Figure 2:**
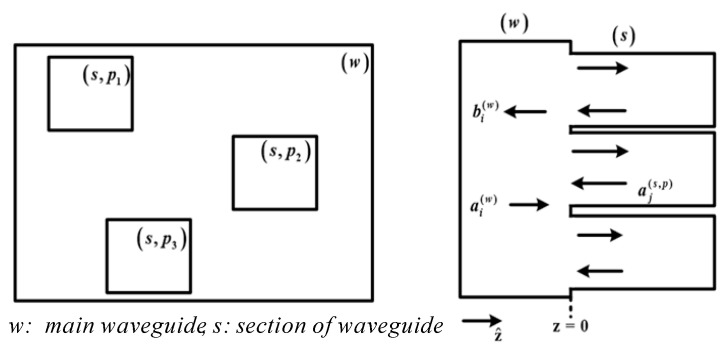
Basic waveguide step with the modal amplitudes incident and scattered by the discontinuity (multiport case) [23].

Firstly, the MMM of the basic waveguide discontinuity is analyzed and derived. As illustrated in Figure 2, this structure represents a multiport case (or *p*-furcation), consisting of a basic waveguide step where modal amplitudes are incident and scattered by the discontinuity [22]. The MMM provides its GSM as a building block for modeling more complex structures, such as the coupler presented in this work. The derivation process follows the approach described in [23] and is briefly summarized here. The transverse electromagnetic fields at the discontinuous plane can be expressed using the mode amplitudes and mode fields of infinite sets of incident modes (*a_i_*) and scattered modes (*b_i_*). The Electric and Magnetic Field Boundary Conditions (EFBC and MFBC, respectively) must be satisfied at the interface between the two discontinuous waveguides. To impose the EFBC and MFBC using the Galerkin method [23], we proceed in two steps: (1) test the EFBC with a generic modal magnetic field ${\vec{h}}_{i}^{w}$ of waveguide (*w*), and (2) test the MFBC with the modal electric fields of ${\vec{e}}_{j}^{s}$ waveguide (*s*). With these definitions, the linear system obtained based on the boundary conditions is expressed as

(1) $$\left\{\begin{array}{l} \mathrm{EFBC}:Q_{w}(a_{w}+b_{w})=\sum_{i=1}^{N}X_{i}^{t}(a_{s,i}+b_{s,i})\\ \mathrm{MFBC}:X_{i}(a_{w}-b_{w})=Q_{s,i}(b_{s,i}-a_{s,i})i=1,2,3,\ldots ,N \end{array}\right.$$

Among them, *Q_w_* and *Q_s,i_* represent the normalized diagonal matrices between the (*w*) region and the (*s*, *i*) region modes, respectively. The *X_i_* represents the coupling coefficient matrix obtained by solving and calculating interactions between various modes. The *a_w_* and *a_s,i_* denote the amplitude of the incident field, and *b_w_* and *b_s,i_* denote the amplitude of the scattered field in each region. The above system can be used to find the values of *b_w_*, *b_s,i_* in terms of *a_w_*, and *a_s,i_*, as in Formula (2):

(2) $$\left\{\begin{array}{l} b_{w}=(I_{w}-2FQ_{w})a_{w}+2F\sum_{i=1}^{N}X_{i}^{t}a_{s,i}\\ b_{s,i}=2Q_{s,i}^{-1}X_{i}FQ_{w}a_{w}+\left(I_{s,i}-2Q_{s,i}^{-1}X_{i}F\sum_{i=1}^{N}X_{i}^{t}\right)a_{s,i} \end{array}\right.$$

where *I_w_* is a *w*-dimensional identity matrix, and *F* is specifically expressed as follows:

(3) $$F={\left(Q_{w}+\sum_{i=1}^{N}X_{i}^{t}Q_{s,i}^{-1}X_{i}\right)}^{-1}$$

Then, this relation (2) is the GSM representation of the waveguide step, and it can be expressed as (4). An expression for the scattered field amplitude in terms of the incident field amplitude is derived, corresponding to each element of the generalized S-parameter matrix.

(4) $$\left[\begin{array}{l} b_{w}\\ b_{s,1}\\ b_{s,2}\\ \vdots \\ b_{s,N} \end{array}\right]=\left[\begin{array}{ccccc} I_{w}-2FQ_{w} & 2FX_{1}^{t} & 2FX_{2}^{t} & \ldots & 2FX_{N}^{t}\\ 2Q_{s,1}^{-1}X_{1}FQ_{w} & I_{s,1}-2Q_{s,1}^{-1}X_{1}FX_{1}^{t} & -2Q_{s,2}^{-1}X_{2}FX_{2}^{t} & \ldots & -2Q_{s,N}^{-1}X_{N}FX_{N}^{t}\\ 2Q_{s,2}^{-1}X_{2}FQ_{w} & -2Q_{s,1}^{-1}X_{1}FX_{1}^{t} & I_{s,2}-2Q_{s,2}^{-1}X_{2}FX_{2}^{t} & \ldots & -2Q_{s,N}^{-1}X_{N}FX_{N}^{t}\\ \vdots & \ldots & \ldots & \ddots & \ldots \\ 2Q_{s,N}^{-1}X_{N}FQ_{w} & -2Q_{s,1}^{-1}X_{1}FX_{1}^{t} & -2Q_{s,2}^{-1}X_{2}FX_{2}^{t} & \ldots & I_{s,N}-2Q_{s,N}^{-1}X_{N}FX_{N}^{t} \end{array}\right]\left[\begin{array}{l} a_{w}\\ a_{s,1}\\ a_{s,2}\\ \vdots \\ a_{s,i} \end{array}\right]$$

**Figure 3:**
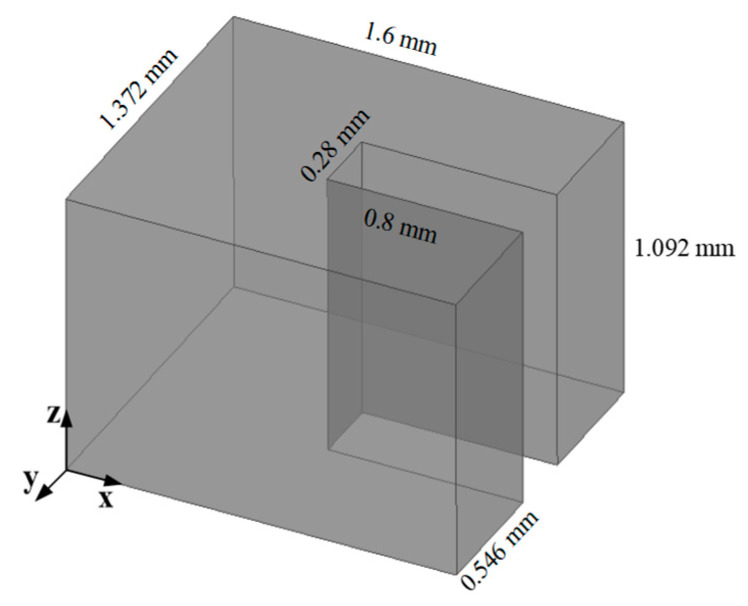
Three-port discontinuous structure (marked dimensions).

**Figure 4:**
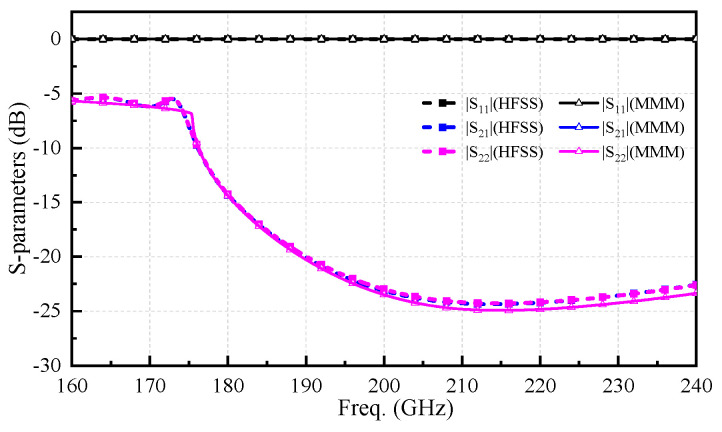
Comparison of calculation results between HFSS and the MMM program for the three-port discontinuous structure.

In this work, the focus is on the three-port discontinuous structure matrix. The relationship between the modal amplitudes and the generalized S-parameters is expressed as follows (5). Subsequently, a MATLAB 2017b program was developed to implement the discussed MMM for calculating the three-port discontinuous structure in Figure 3. This three-port structure serves as a basic constituent unit of the waveguide coupler structure in Figure 1, based on the MMM calculation. The S-parameters of this three-port discontinuity, computed using the MMM-based program, are compared with those simulated from the commercial software Ansys HFSS, as shown in Figure 4. The accuracy of the MMM is highly dependent on the number of retained higher-order modes. To verify the convergence of the MMM formulation, a study on the impact of mode number *N* was conducted. It was found that the S-parameters stabilize once *N* exceeds 30. For instance, at the center frequency, there is no significant difference in |S_22_| between *N* = 30 and *N* = 50, confirming that *N* = 30 provides sufficient accuracy for the analysis of the stacked coupler. The results are in highly consistent agreement regarding transmission, coupling, and reflection characteristics across the wideband of 160–240 GHz. This comparison validates the feasibility and accuracy of the developed MMM-based program for analyzing three-port discontinuous waveguide structures.

(5) $$\left[\begin{array}{c} b_{w}\\ b_{s,1}\\ b_{s,2} \end{array}\right]=\left[\begin{array}{ccc} I_{w}-2FQ_{w} & 2FX_{1}^{t} & 2FX_{2}^{t}\\ 2Q_{s,1}^{-1}X_{1}FQ_{w} & I_{s,1}-2Q_{s,1}^{-1}X_{1}FX_{1}^{t} & -2Q_{s,2}^{-1}X_{2}FX_{2}^{t}\\ 2Q_{s,2}^{-1}X_{2}FQ_{w} & -2Q_{s,1}^{-1}X_{1}FX_{1}^{t} & I_{s,2}-2Q_{s,2}^{-1}X_{2}FX_{2}^{t} \end{array}\right]\left[\begin{array}{c} a_{w}\\ a_{s,1}\\ a_{s,2} \end{array}\right]$$

### 2.3. MMM Analysis for Coupler

Secondly, the traditional waveguide branch coupler can be structured by cascading the afore-mentioned waveguide discontinuities. The fundamental generalized S-parameters cascading theory is finally used to realize the mode-matching analysis of the overall network branch coupler. A corresponding MATLAB program has been developed to implement this MMM-based approach.

A critical advantage of utilizing GSM cascading over traditional transmission matrix multiplication is the preservation of numerical stability. In a complex nine-layer stacked architecture, cascading transmission matrices often lead to severe matrix ill-conditioning due to the unconstrained exponential growth of evanescent modes. GSM cascading intrinsically bypasses this issue by calculating bounded reflection and transmission wave amplitudes, thereby preventing cumulative numerical errors and ensuring robust stability across the entire multi-layer network. Additionally, standard relative convergence criteria are strictly observed during mode truncation to suppress numerical artifacts at the discontinuous boundaries.

**Figure 5:**
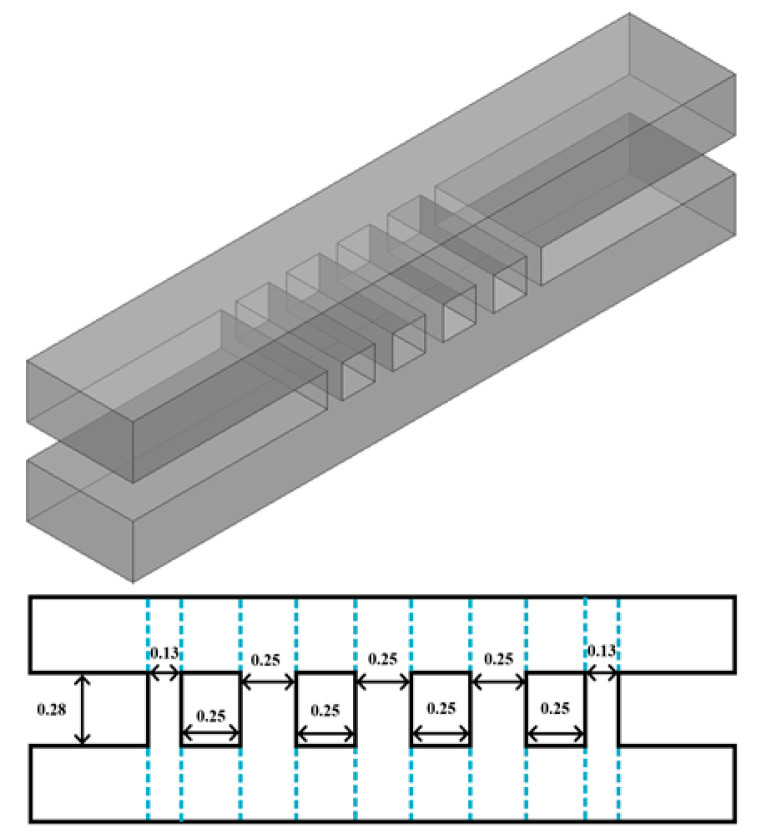
The proposed five-branch coupler with size marking.

**Figure 6:**
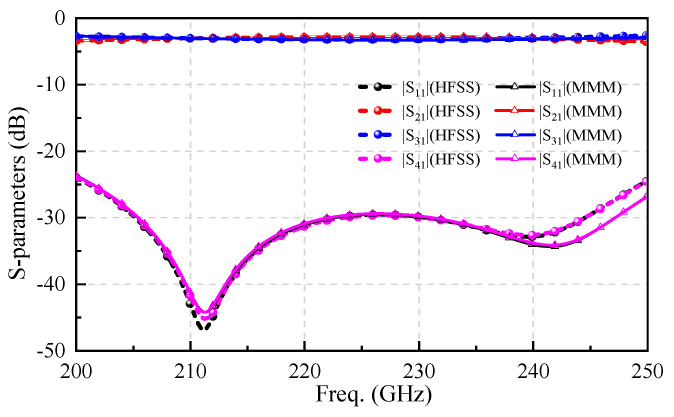
Comparison of calculation results between HFSS and the MMM program for the five-branch coupler.

To validate the accuracy of the MMM algorithm in calculating the multi-branch waveguide coupler, a five-branch waveguide coupler operating in the 220 GHz band was designed based on traditional coupler theory, as shown in Figure 5, with key parameter dimensions annotated in millimeters. The S-parameters of such a five-branch waveguide coupler have been obtained through the MMM program and are compared with those simulated from Ansys HFSS, as shown in Figure 6. Obviously, within the range of 200–250 GHz, the MMM calculated results are highly consistent with those in Ansys HFSS, with an accuracy rate close to 100%. Both methods indicate that the return loss of the proposed coupler remains better than −25 dB throughout the operational band.

It is worth mentioning that the program we developed directly records the time taken in the calculation process by loading the timing function pair (toc/tic). For HFSS, timing is based on results at the beginning of its operation and at the end of the analysis. The solution process was optimized using adaptive mesh refinement with a maximum ΔS set to 0.02. Comparative results show that the MMM requires only 6 s, whereas the HFSS simulation takes 75 s. Both the MMM algorithm and the HFSS simulations were executed on the same personal computer, equipped with an Intel Core i5-11400 processor and 16 GB of RAM. The MMM program consumed a peak memory allocation of 42 MB, while the equivalent HFSS model required 2.4 GB of memory to achieve a converged mesh. While ensuring accurate results for waveguide couplers, the proposed method achieves an order-of-magnitude reduction in calculation time. This significant improvement greatly enhances the efficiency of calculating and analyzing THz waveguide components, demonstrating considerable practical value for applications involving extensive iterative simulations and statistical error analysis [24,25].

The computational overhead of the proposed MMM is primarily governed by the number of truncated modes. There is a direct cubic correlation between the mode count and the execution time, stemming from the matrix inversion processes required at each waveguide junction. In practice, this correlation manifests as a trade-off between numerical precision and simulation speed. For the 220 GHz waveguide structure investigated in this work, approximately 30 modes were found to be sufficient to achieve stable results, with further increases producing negligible variation in the computed S-parameters. This mode truncation level, therefore, provides a good balance between computational accuracy and efficiency.

## 3. Fabrication, Packaging and Measurement

**Figure 7:**
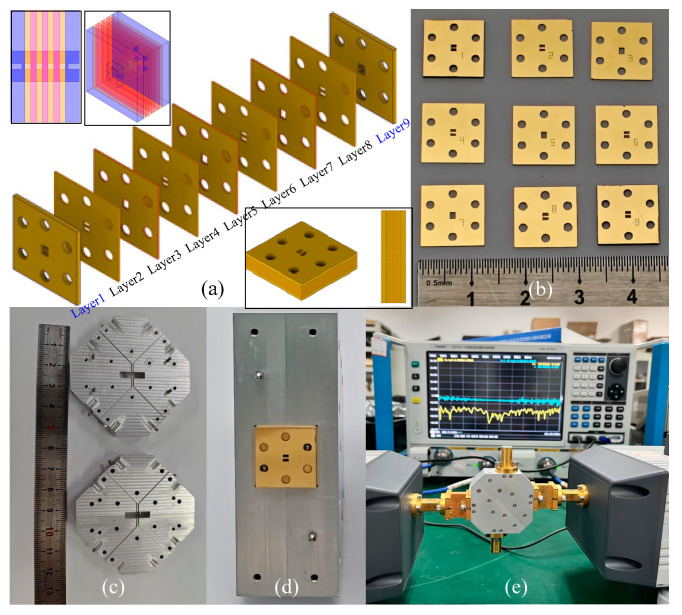
Photographs of (**a**) the coupled layered stacking structure (the inset shows the coupler structure and the stacked coupler), (**b**) the metalized silicon platelets with 9 layers, (**c**) the block machined by CNC, (**d**) the block equipped with a coupler chip, and (**e**) the measurement setup.

The five-branch coupler, utilizing WR-4.3 standard input/output waveguides (1.092 × 0.546 mm) at all ports, would be fabricated using advanced DRIE technology. The waveguide coupler can be split into nine layers with the cross-sectional positions at layers 2–8, respectively, as shown in Figure 1. Step-etching is employed to create features with a depth of 0.13 mm in layers 1 and 9, which are fabricated from a 0.635 mm-thick wafer. The remaining layers utilize through-hole etching technology and are fabricated from 0.25 mm-thick wafers, as shown in Figure 7a. Nine square silicon platelets, each measuring 12 mm × 12 mm, are employed in the device. To minimize misalignment, six through-holes with a diameter of 150 μm are etched into each platelet for alignment purposes. Further gold plating will introduce an additional 2 µm thickness for each plane of every silicon platelet, as seen in Figure 7b. More detailed information on the DRIE technology processes can be found in our previous work [9].

The final stackable multilayer packaging technology will be adopted to complete the full coupler structure. The packaged coupler has dimensions of *w × l × h* = 12 mm × 12 mm × 3 mm, as shown in Figure 7a,d. It is evident that the proposed coupler features a compact architecture, with a total thickness of only 3 mm. This dimension is substantially smaller than that of waveguide couplers produced by traditional CNC machining. Moreover, the design is better suited for multi-layer stacked THz systems, allowing integration with other multi-functional devices. Additionally, a dedicated holder must be fabricated to assemble the coupler chip, facilitating a solid structure for accurate measurements. As shown in Figure 7c, an aluminum holder was manufactured using the E-plane split technique. The central cavity of this holder is precision-machined to perfectly match the coupler chip. Curved waveguide transitions connect all four input and output ports to the surface of the block, where standard UG-387 flanges are installed. Figure 7d shows the fully assembled block equipped with the integrated coupler chip. The volume of the packaged assembly is *W* × *L* × *H* = 50 × 50 × 20 mm.

S-parameter measurements of this device under test (DUT), which incorporates the coupler chip, are carried out using a Ceyear 3672E (Ceyear Technologies Co., Ltd., Qingdao, China) Vector Network Analyzer (VNA) equipped with WR-4.3 band frequency extension modules after through-reflect-line (TRL) calibration. The TRL calibration procedure involves three steps: (1) Establish the thru connection using Shim #1 between the two converters to perform the “Thru” calibration. (2) Connect the Short standard to frequency converter-1 to initiate the “Reflect” calibration, and repeat the process using the Short at converter-2. (3) Establish the line connection using Shim #2 between the ports to complete the “Line” calibration. All shims and standards are available from the Ceyear WR-4.3 calibration kit. Then, the DUT is connected to standard WR-4.3 waveguide converters via UG-387 flanges and fixed by screws, as illustrated in the measurement setup in Figure 7e. Since the coupler is a reciprocal four-port device, only the through, coupled, and isolated S-parameters require measurement to fully characterize its performance. The reflection coefficient can be derived from any of these measurements. Due to the availability of only two frequency extenders, the assembly is measured in three distinct configurations, with any unmeasured ports terminated using matched waveguide loads.

## 4. Results Discussion and Analysis

**Figure 8:**
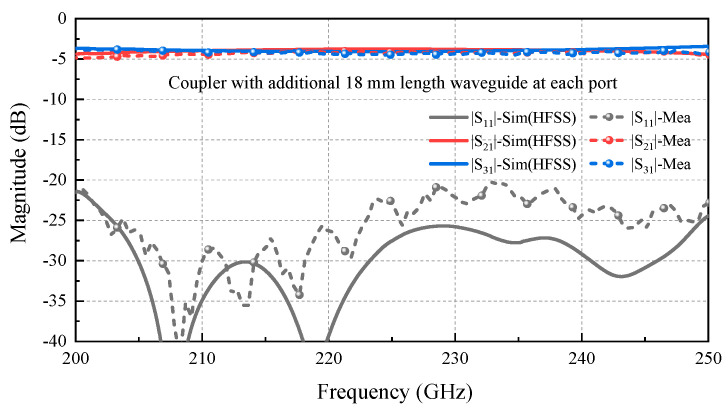
Performance comparison between the measurement and simulation of the proposed coupler DUT.

**Figure 9:**
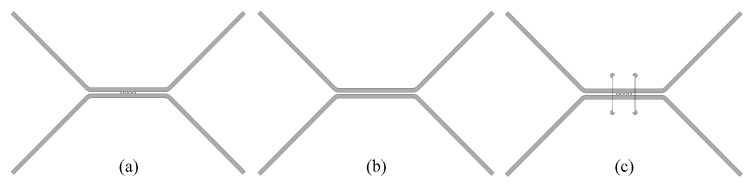
Three different simulation models: (**a**) a coupler with a long straight waveguide at each port; (**b**) two waveguide structures with straight waveguides of the same length as in (**a**); and (**c**) a complicated coupler with air gaps between the chip and long straight waveguides, referencing the size of the block in Figure 7c.

Figure 8 presents the measured S-parameter of the holder assembly integrated with the coupler chip, along with a comparison to simulation results. This simulated coupler model incorporates 18 mm-long straight waveguides to match the physical length of the extender waveguides in the Figure 7c holder, as depicted in Figure 9a. Additionally, degraded aluminum with an equivalent conductivity of 1 × 10^7^ S/m (about a quarter of the theoretical conductivity value of 3.8 × 10^7^ S/m) is used in all the extended straight waveguide models. This adjusted value accounts for multiple effects, including non-ideal aluminum material, surface roughness and imperfect electric contact resulting from CNC machining, as well as other potential issues introduced during machining, assembly, and testing processes [26]. The acquisition of this equivalent conductivity value will be discussed in detail in Section 4.1. The boundary of the main coupler model (approximately 3 mm in length) is modeled as gold with a theoretical conductivity of 4.1 × 10^7^ S/m. As shown in Figure 8, the measured performance of the coupler sample agrees well with the simulation results of the model introduced above in Figure 9a, including power distribution and return loss. Moreover, the isolation performance exhibits consistency with the return loss characteristics. In the band from 200 GHz to 250 GHz, the power can be evenly divided, and the return loss is better than −20 dB. Above satisfactory results indicate that the CNC-machined block holder aligns well with the 3D-stacked coupler. However, issues such as misalignment-induced gaps remain, leading to slightly higher measured insertion loss (IL) compared to simulations. A more detailed analysis of ILs is presented in the following Section 4.1.

On the other hand, machining and assembly errors, such as etching depth errors from the DRIE process, non-uniform silicon layer thickness, and misalignment errors from 3D stacked packaging, are crucial problems in such layered couplers. As a result, our proposed MMM facilitates sufficient uncertainty analysis for such layered couplers, substantially reducing the time required for performance evaluation under manufacturing errors. This work analyzes the performance uncertainty of the coupler under manufacturing variations, including etching depth errors of ±20 μm, silicon thickness deviations of ±20 μm, and misalignment tolerances of ±10 μm. It is worth highlighting that only the statistical distribution of quantified maximum amplitude imbalance (∆A) and absolute bandwidth (∆B) characteristics, as quantified by the MMM program, are presented. In this Monte Carlo analysis, fabrication variables, including etching depth, wafer thickness, and alignment, are assumed to be independent and uncorrelated. This assumption is justified by the distinct nature of DRIE, substrate sourcing, and bonding processes. Treating these as uncorrelated variables allows for a conservative evaluation of the coupler’s performance robustness, accounting for the random nature of multi-layer 3D integration. Quantified maximum amplitude imbalance is defined as max (|S21–S31|), and the quantified absolute bandwidth is defined as |S21| and |S31| within the scope of −3 dB ± 0.25 dB.

### 4.1. Extremely Low-Loss Characteristic

**Figure 10:**
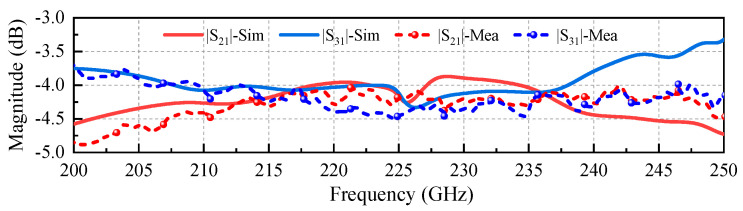
Insertion loss: measurement (block holder) versus simulation. (model in Figure 9c).

**Figure 11:**
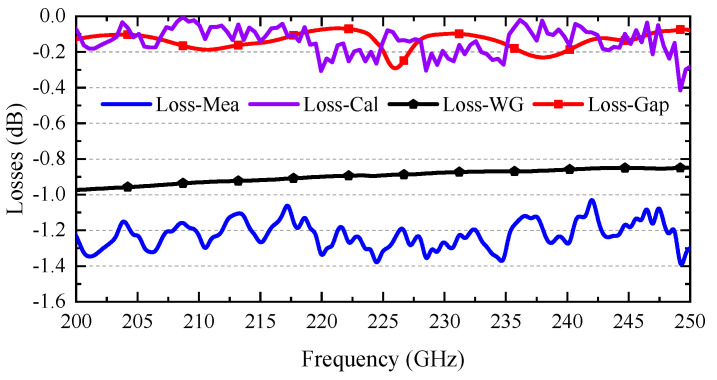
Loss performance across different structures.

Figure 10 presents a detailed comparison between the measured and simulated ILs of the coupler holder DUT. The simulated model (Figure 9c) incorporates a detailed representation of the coupler, including air gaps between the chip and the straight waveguides to reflect actual assembly conditions. The results are consistent, confirming the rationality and accuracy of the modeling approach. To better express the loss characteristics of the coupler, including two outputs, we define the insertion loss as: Loss = 10 log_10_ [(P2 + P3)/P1]. Therefore, the measured coupler holder DUT loss performance of approximately −1.3 dB is displayed in Figure 11.

As previously indicated, current measurements only allow characterization of the total loss of the entire assembled block (as shown in Figure 8), rather than that of the coupler chip itself. To account for the individual loss of the packaged coupler chip, we propose a three-step calibration method that integrates simulation with experimental measurements to extract the loss characteristics specifically attributable to the packaged coupler chip.

#### 4.1.1. Calibration of Rectangular Waveguides

Firstly, an additional aluminum block, containing only straight waveguides based on the same material and CNC process, has been fabricated. The waveguide structure includes a model, shown in Figure 9b, which has the same length of waveguide sections as the block holder in Figure 9a. After measuring this block and comparing the results with simulation data, an equivalent conductivity of 1 × 10^7^ S/m is derived. In the three-step loss calibration method, the aluminum block is assigned an equivalent conductivity of 1 × 10^7^ S/m. The skin depth at THz frequencies is extremely shallow and comparable to the surface roughness introduced by the fabrication process. Consequently, scattering from surface roughness, native oxide layers, and imperfect mechanical contact between the stacked layers collectively degrade effective conductivity. Such equivalent conductivity modeling has been widely adopted in THz waveguide measurements to accurately represent real-world loss conditions. The extracted conductivity value, therefore, provides a reasonable representation of the effective electrical behavior of the aluminum block used in the measurement setup. This value comprehensively accounts for the effects of various unfavorable factors during machining, assembly, and testing on waveguide loss. The simulated loss for such a block, ranging from −0.8 to −1 dB, is presented in Figure 11. This loss, attributable to the extended waveguide sections, constitutes the primary source of loss in the coupler holder assembly.

#### 4.1.2. Calibration of the Gap Between Chip and Block

Secondly, an equivalent conductivity of 1 × 10^7^ S/m has been applied to all straight waveguides in the simulation model depicted in Figure 9c. Two air gaps have also been introduced between the coupler chip and the extension waveguide to simulate the case as it would be in actual assembly. After comparing the simulation loss with measurement data, we can confirm the presence of the two gaps, each 10 µm in size. Furthermore, through calibrating the waveguide loss, the additional loss attributable to each air gap is estimated to be approximately −0.2 dB, as shown in Figure 11. It should be noted that the additional loss of approximately −0.2 dB per 10 μm gap was not obtained from an independent direct measurement of a single gap. Instead, it was extracted through the three-step calibration procedure described above. After the total loss of the assembled holder was measured, the loss of the straight extension waveguides was first calibrated by comparing the reference waveguide block with the simulation. The remaining excess loss in the assembled model containing two chip-to-block air gaps was then attributed to the gap discontinuities. The reported gap loss should be understood as a calibration-based estimate obtained from combined simulation and measurement. In addition, the extracted −0.2 dB value corresponds to the specific assembled case with a 10 μm gap in this work. The gap dimension affects the discontinuity strength between the coupler chip and the extension waveguide. A smaller gap, such as 5 μm, is expected to introduce lower excess insertion loss, while a larger gap, such as 20 μm, would lead to stronger mismatch and higher additional loss. This trend indicates that the gap-induced loss is geometry-dependent rather than a fixed constant. Nevertheless, within the reasonable assembly tolerance range, the extracted intrinsic loss of the coupler remains in the ultra-low-loss regime, which supports the validity of the main conclusion.

#### 4.1.3. Acquisition of Coupler Chip Loss

Thirdly, by applying the defined loss calculation formula and calibrating the waveguide and gap losses, the individual loss contribution of the packaged coupler chip can be estimated. The extracted results indicate that this 3D-stacked multilayer coupler, with a total thickness of 3 mm, exhibits an intrinsic loss of less than −0.2 dB, with an average estimated value of −0.14 dB. To the author’s knowledge, this represents the lowest loss reported to date for a coupler operating in this frequency band. These results validate the advanced nature of the proposed design methods, fabrication processes, and assembly techniques.

### 4.2. Analysis of Etching Depth Error

**Figure 12:**
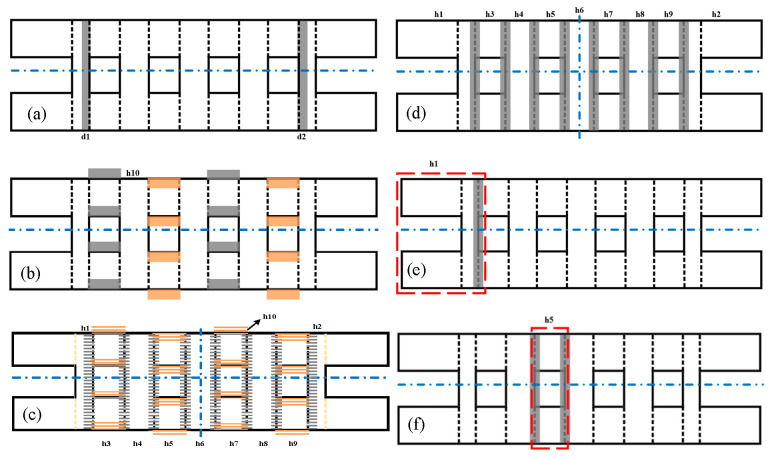
The manifestation forms of different errors in terms of coupler structure are: (**a**) etching depth errors; (**b**) misalignment errors; (**c**) comprehensive errors; (**d**) silicon wafer thickness errors, (**e**) typical h1 error, and (**f**) h5 error.

As mentioned above, the proposed MMM enables further analysis of the impact of uncertainty errors on the coupler performance, such as the etching depth (d1 and d2) in Figure 12a. To evaluate the performance, the MMM was employed to investigate the maximum amplitude imbalance (∆A) and absolute bandwidth (∆B), with subsequent analysis of their distribution. This process can be performed in three straightforward steps.

**Figure 13:**
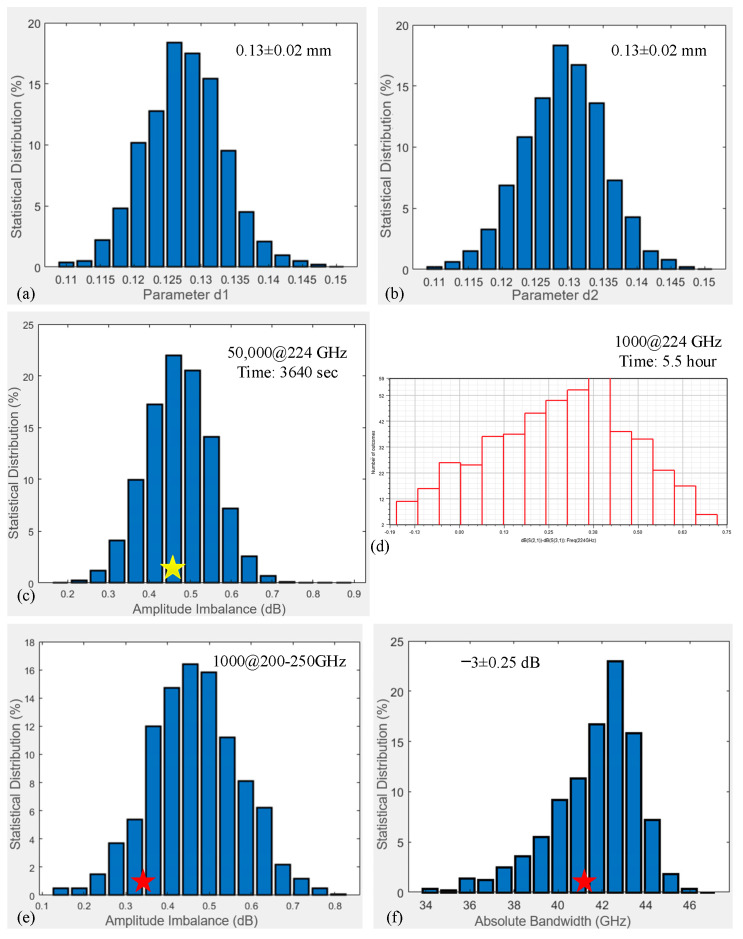
Statistical distribution chart of errors and performance impacts of etching depth d1 or d2: (**a**) d1 error distribution, (**b**) d2 error distribution, (**c**) 50,000 ∆A simulations at 224 GHz, (**d**) 1000 ∆A simulations at 224 GHz using HFSS, (**e**) 1000 ∆A simulations across 200–250 GHz, and (**f**) 1000 ∆B simulations across 200–250 GHz.

The Monte Carlo method [27] is used to generate etching depth parameter errors with specific distributions, as shown in Figure 13a,b. The rule-of-thumb value 130 ± 20 μm is applied to both errors, and the corresponding uncertainty parameters are described by a truncated normal distribution [28]. A worst-case scenario using a uniform distribution over the ±20 μm tolerance range would lead to a wider spread in amplitude imbalance (ΔA) and a potential reduction in the absolute bandwidth (ΔB), as it assigns a higher probability to extreme fabrication offsets. Subsequently, 50,000 simulations are pre-conducted at the center frequency of 224 GHz here. The ∆A distribution at 224 GHz is statistically obtained, as shown in Figure 13c. The yellow star marks the simulated ΔA value of 0.45 dB at 224 GHz. Within the range of 0.2–0.9 dB, the ∆A performance is characterized by a truncated normal distribution. The proportion of statistical times within the range of 0.3–0.6 dB is over 80%, which is consistent with the conclusion that the maximum ∆A of branch couplers is around the center frequency. It is worth emphasizing that this MMM requires a mere 3640 s, which is already far less than the 5.5 h consumed by 1000 HFSS simulation calculations at Figure 13d. A total of 1000 simulation calculations is performed to determine the distribution of maximum ∆A over the 200–250 GHz band, as shown in Figure 13e. The analysis results suggest that the data exhibit a truncated normal distribution within the range of 0.1–0.8 dB. The distribution of ∆B in Figure 13f reveals an inverse relationship with the loading error. Most of the data points lie within the 36–46 GHz band.

From the calibrated results of the coupler chip discussed previously, the maximum ∆A exhibits a value of 0.32 dB within the absolute bandwidth of 41.6 GHz. Both calibrated values are marked with red stars on the error distribution graphs in Figure 13e,f, demonstrating normal distribution characteristics.

### 4.3. Analysis of Silicon Wafer Thickness Error

**Figure 14:**
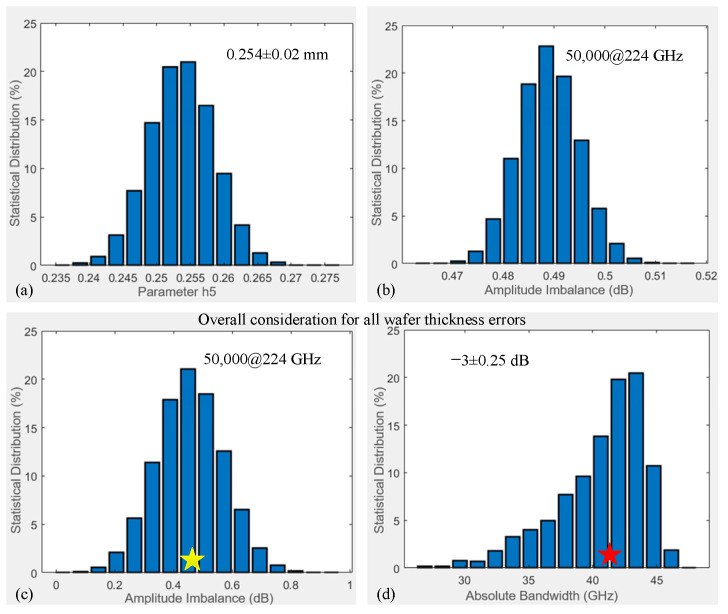
Statistical distribution chart of errors and performance impacts of silicon wafer thicknesses: (**a**) h5 error distribution, (**b**) 50,000 ∆A simulations at 224 GHz with only h5 error, (**c**) 50,000 ∆A simulations at 224 GHz, with overall consideration for all thickness errors, and (**d**) 1000 ∆B simulations, considering all thickness errors, across 200–250 GHz.

In this section, silicon wafer thickness errors and their impact on the coupler performance of ∆A and ∆B are discussed. First, the error structures corresponding to all silicon wafer thicknesses are illustrated with shaded areas in Figure 12d, employing the variables h1…h9 for representation. Typically, silicon wafer thickness errors, h1 (0.635 mm) and h5 (0.25 mm), are provided in Figure 12e,f here. The rest are similar and not displayed one by one here. Second, classic parameter h5 errors are generated based on the Monte Carlo method, as shown in Figure 14a. All thickness errors can be created using the Monte Carlo method, which is characterized by a truncated normal distribution confined to ±20 μm. Third, 50,000 simulations of ∆A are calculated based on the MMM at 224 GHz and statistically obtained, as distributed in Figure 14b. While this error induces a greater overall ∆A, the distribution remains consistent with a truncated normal form. As the remaining thickness errors yield analogous results, they are not detailed individually here.

In particular, considering all h1…h9 errors and evaluating the coupler performance is highly interesting. Figure 14c displays the ∆A distribution from 50,000 simulations at 224 GHz, which follows a truncated normal distribution. The ∆B distribution in Figure 14d reveals a similar inverse relationship with this type of loading error. The majority of the data fall within the 30–46 GHz range. As denoted by a red star in Figure 14d, the calibrated maximum ∆A of 0.32 dB falls within the simulated error distribution, validating the measurement accuracy.

### 4.4. Analysis of Misalignment Error

**Figure 15:**
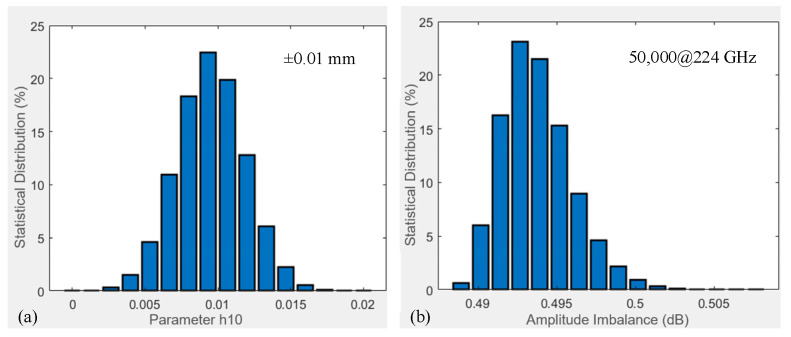
Statistical distribution chart of errors and performance impacts of misalignments: (**a**) h10 error distribution, (**b**) 50,000 ∆A simulations at 224 GHz.

In this section, misalignment errors caused during the assembly process and their impact on coupler ∆A characteristics are developed. The shaded areas in Figure 12b indicate the manifestation of misalignment error on the coupler structure, represented by h10. Parameter h10 errors also exhibit a truncated normal distribution within the range of ±10 μm, as shown in Figure 15a. A total of 50,000 simulations of ∆A are performed using the MMM at 224 GHz, with the results statistically analyzed and plotted in Figure 15b. While the observed ∆A values are generally larger under this error condition, their distribution remains truncated normal. Similar trends are observed for other misalignment errors and ∆B characteristics, which are omitted here for brevity.

### 4.5. Generalized Analysis of All Errors

In previous analyses, the effects of three distinct error types on coupler performance were evaluated individually, including ±20 μm etching depth errors, ±20 μm silicon thickness errors, and ±10 μm misalignment errors. The primary sources of residual measurement uncertainty include test-port cable flexure during DUT connection, minor ambient temperature fluctuations affecting the multipliers in the extension modules, and microscopic mechanical misalignments at the WR-4.3 waveguide flange interfaces. These factors combined contribute to a slight measurement tolerance band, which accounts for the minor deviations observed between the nominal simulated responses and the measured S-parameters. However, multiple fabrication and assembly errors often coexist during the manufacturing of such 3D-stacked coupler chips. Therefore, in this section, all errors and their combined impact on coupler performance will be comprehensively considered, as well as statistical analysis of ∆A and ∆B will be conducted across 200–250 GHz.

**Figure 16:**
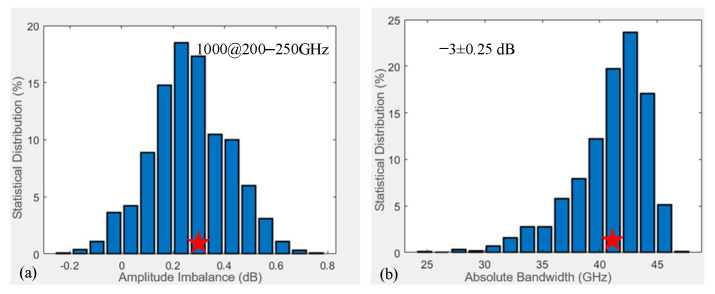
Statistical distribution chart of errors and performance impacts (all etching depth, wafer thickness and misalignment errors are considered): (**a**) 1000 ∆A simulations, and (**b**) 1000 ∆B simulations, across 200–250 GHz.

The shaded regions in Figure 12c can represent the expression of all error types on the coupler structure. The next step can involve using a similar Monte Carlo method to generate error sets for all variable parameters that represent the truncated normal distribution. Similar to previous figures (Figure 13a,b and Figure 14a), these plots are not reproduced here. Finally, a total of 1000 simulations is conducted to determine the distribution of the maximum ∆A across the 200–250 GHz band, as illustrated in Figure 16a. The analysis indicates a truncated normal distribution in the range of 0–0.6 dB. Similarly, Figure 16b displays the ∆B distribution, demonstrating a reduction under loading error conditions, with most values concentrated between 30 and 46 GHz. Additionally, the calibrated maximum ∆A of 0.32 dB and ∆B of 41.6 GHz have been marked with red stars in Figure 16a and b, respectively, demonstrating the accuracy, efficiency, and reliability of this proposed MMM-based uncertainty analysis approach.

All results discussed above collectively indicate that this highly integrated branch coupler has successfully achieved the desired performance through stackable multilayer DRIE technology, notably exhibiting state-of-the-art loss performance below −0.2 dB. Furthermore, the proposed MMM enables rapid computation, uncertainty analysis, and statistical evaluation of the waveguide coupler, offering orders-of-magnitude faster processing compared to the conventional finite-element method (FEM) used in HFSS.

## 5. Conclusions

This article has developed an accurate and efficient MMM for the fast analysis of branch waveguide couplers, offering orders-of-magnitude speed improvement compared with the traditional method using the FEM in HFSS. This MMM has been achieved by cascading the generalized S-parameters of two-port and three-port waveguide discontinuities. It is the best candidate for fast analysis of extensive uncertainty errors in branch waveguide couplers fabricated using a 3D silicon-based stacking process. The MMM is optimized for rectangular step discontinuities like the stacked coupler in this work; its efficiency decreases for complex geometries. Curved structures require ‘staircasing’ approximations that increase computation and potential errors, while arbitrary cross-sections necessitate numerical 2D eigensolvers to extract modes. The theoretical work has been validated by calculating, characterizing, and analyzing the uncertainty of a DRIE-fabricated and 3D-stacked 220 GHz five-branch waveguide coupler. This 3D-stacked multilayer coupler, with a thickness of only 3 mm, has achieved an extremely low-loss performance of less than −0.2 dB. While the validation in this study is primarily demonstrated on a 220 GHz five-branch coupler, the proposed MMM framework is inherently scalable and highly generalizable to higher frequencies, given that the semi-analytical MMM formulation relies on the exact matching of boundary conditions, it scales proportionally with the wavelength and waveguide dimensions without algorithmic modification. In addition, this approach can be extended to a wide range of applications involving rapid computation and uncertainty assessment for various 3D- stacked multilayer waveguide components, particularly those used in next-generation THz transmitters and receivers based on 3D stacked packaging technology.

## Data Availability

The data and core algorithm workflow presented in this study are available on reasonable request from the corresponding author.
